# A Preliminary Checklist (METRICS) to Standardize the Design and Reporting of Studies on Generative Artificial Intelligence–Based Models in Health Care Education and Practice: Development Study Involving a Literature Review

**DOI:** 10.2196/54704

**Published:** 2024-02-15

**Authors:** Malik Sallam, Muna Barakat, Mohammed Sallam

**Affiliations:** 1 Department of Pathology, Microbiology and Forensic Medicine School of Medicine The University of Jordan Amman Jordan; 2 Department of Clinical Laboratories and Forensic Medicine Jordan University Hospital Amman Jordan; 3 Department of Translational Medicine Faculty of Medicine Lund University Malmo Sweden; 4 Department of Clinical Pharmacy and Therapeutics Faculty of Pharmacy Applied Science Private University Amman Jordan; 5 Department of Pharmacy Mediclinic Parkview Hospital Mediclinic Middle East Dubai United Arab Emirates

**Keywords:** guidelines, evaluation, meaningful analytics, large language models, decision support

## Abstract

**Background:**

Adherence to evidence-based practice is indispensable in health care. Recently, the utility of generative artificial intelligence (AI) models in health care has been evaluated extensively. However, the lack of consensus guidelines on the design and reporting of findings of these studies poses a challenge for the interpretation and synthesis of evidence.

**Objective:**

This study aimed to develop a preliminary checklist to standardize the reporting of generative AI-based studies in health care education and practice.

**Methods:**

A literature review was conducted in Scopus, PubMed, and Google Scholar. Published records with “ChatGPT,” “Bing,” or “Bard” in the title were retrieved. Careful examination of the methodologies employed in the included records was conducted to identify the common pertinent themes and the possible gaps in reporting. A panel discussion was held to establish a unified and thorough checklist for the reporting of AI studies in health care. The finalized checklist was used to evaluate the included records by 2 independent raters. Cohen κ was used as the method to evaluate the interrater reliability.

**Results:**

The final data set that formed the basis for pertinent theme identification and analysis comprised a total of 34 records. The finalized checklist included 9 pertinent themes collectively referred to as METRICS (Model, Evaluation, Timing, Range/Randomization, Individual factors, Count, and Specificity of prompts and language). Their details are as follows: (1) Model used and its exact settings; (2) Evaluation approach for the generated content; (3) Timing of testing the model; (4) Transparency of the data source; (5) Range of tested topics; (6) Randomization of selecting the queries; (7) Individual factors in selecting the queries and interrater reliability; (8) Count of queries executed to test the model; and (9) Specificity of the prompts and language used. The overall mean METRICS score was 3.0 (SD 0.58). The tested METRICS score was acceptable, with the range of Cohen κ of 0.558 to 0.962 (*P*<.001 for the 9 tested items). With classification per item, the highest average METRICS score was recorded for the “Model” item, followed by the “Specificity” item, while the lowest scores were recorded for the “Randomization” item (classified as suboptimal) and “Individual factors” item (classified as satisfactory).

**Conclusions:**

The METRICS checklist can facilitate the design of studies guiding researchers toward best practices in reporting results. The findings highlight the need for standardized reporting algorithms for generative AI-based studies in health care, considering the variability observed in methodologies and reporting. The proposed METRICS checklist could be a preliminary helpful base to establish a universally accepted approach to standardize the design and reporting of generative AI-based studies in health care, which is a swiftly evolving research topic.

## Introduction

The integration of generative artificial intelligence (AI) models into health care education and practice holds promising perspectives with numerous possibilities for continuous improvement [1-5]. Examples of generative AI-based conversational models characterized by ease of use and perceived usefulness include ChatGPT by OpenAI, Bing by Microsoft, and Bard by Google [6-8].

The vast potential of generative AI-based models in health care can be illustrated as follows. First, generative AI-based models can facilitate streamlining of the clinical workflow, with subsequent improvement in efficiency manifested in reduced time for care delivery and reduced costs [1,9-11]. Second, generative AI-based models can enhance personalized medicine with a huge potential to achieve refined prediction of disease risks and outcomes [1,12,13]. Third, generative AI-based models can be implemented to improve health literacy among lay individuals through the provision of easily accessible and understandable health information [1,14,15].

Despite the aforementioned advantages of generative AI-based models in health care, several valid concerns were raised, which should be considered carefully owing to their serious consequences [1,4,16]. For example, the lack of clarity on how generative AI-based models are trained raises ethical concerns [17,18]. Additionally, these models have an inherent bias in the generated content based on the modality of training used for their development and updates [17,18]. Importantly, the generation of inaccurate or misleading content, which might appear scientifically plausible to nonexperts (referred to as “hallucinations”), could have profound negative impacts in health care settings [1,19-21]. Furthermore, the integration of generative AI-based models in health care could raise complex medicolegal and accountability questions, compounded by the issues of data privacy and cybersecurity risks [1,4,22,23].

Similarly, the use of generative AI-based models can cause a paradigm shift in information acquisition, particularly in health care education [1,24-26]. However, careful consideration of the best policies and practices to incorporate AI-based models in health care education is needed [27]. This issue involves the urgent need to address the issues of inaccuracies, possible academic dishonesty, decline in critical thinking development, and deterioration of practical training skills [1].

Recently, a remarkable number of studies investigated the applicability and disadvantages of prominent generative AI-based conversational models, such as ChatGPT, Microsoft Bing, and Google Bard, in various health care and educational settings [1,2,4,28-34]. However, synthesizing evidence from such studies can be challenging owing to several reasons. Variations in methodologies implemented in various studies as well as in the reporting standards is a major limitation. This issue could hinder the efforts aiming to compare and contrast the results of generative AI-based studies in health care, contributing to the complexity in this domain. This variability arises from several factors, including different settings of the tested models, prompt variability, varying approaches used to evaluate the generated content of generative AI-based models, varying range of tested topics, and possible bias in selecting the tested subjects. Additionally, variability can be related to the different number and varying expertise of individual raters of content quality, as well as the variable number of queries executed, among other factors [35-37].

Therefore, it is important to initiate and develop an approach that can aid in establishing standardized reporting practices for studies aiming to evaluate the content of generative AI-based models, particularly in health care. This standardization can be crucial to facilitate the design of generative AI-based studies in health care, ensuring rigor and achieving precise comparison and credible synthesis of findings across different studies. Thus, we aimed to propose a preliminary framework (checklist) to establish proper guidelines for the design and reporting of findings of generative AI-based studies that address health care–related topics. 

## Methods

### Study Design

The study was based on a literature review to highlight the key methodological aspects in studies that investigated 3 generative AI-based models (ChatGPT, Bing, and Bard) in health care education and practice. The literature review was conducted to identify relevant literature indexed in databases up to November 11, 2023 [38]. The databases used for this literature search were Scopus, PubMed/MEDLINE, and Google Scholar.

### Ethical Considerations

This study did not involve human subjects, and thus, the requirement of ethical permission was waived.

### Literature Search to Identify Relevant Records

The Scopus string query was as follows: (TITLE-ABS-KEY(“artificial intelligence” OR “AI”) AND TITLE-ABS-KEY (“healthcare” OR “health care”) AND TITLE-ABS-KEY (“education” OR “practice”)) AND PUBYEAR > 2022 AND DOCTYPE (ar OR re) AND (LIMIT-TO (PUBSTAGE , “final”)) AND (LIMIT-TO (SRCTYPE , “j”)) AND (LIMIT-TO (LANGUAGE , “English”)). The Scopus search yielded a total of 843 documents.

The PubMed advanced search tool was used as follows: (“artificial intelligence”[Title/Abstract] OR “AI”[Title/Abstract]) AND (“healthcare”[Title/Abstract] OR “health care”[Title/Abstract]) AND (“education”[Title/Abstract] OR “practice”[Title/Abstract]) AND (“2022/12/01”[Date - Publication] : “2023/11/11”[Date - Publication]). The PubMed search yielded a total of 564 records.

In Google Scholar and using the Publish or Perish software (version 8), in the title words and in the years 2022-2023, the search was as follows: “artificial intelligence” OR “AI” AND “healthcare” OR “health care” AND “education” OR “practice,” with a maximum of 999 records retrieved [39].

### Criteria for Record Inclusion

The records from the 3 databases were merged using EndNote 20.2.1 software. This was followed by removal of duplicate records and removal of preprints by using the following function: ANY FIELD preprint OR ANY FIELD rxiv OR ANY FIELD SSRN OR ANY FIELD Researchgate OR ANY FIELD researchsquare OR ANY FIELD. The retrieved records were eligible for the final screening step given the following inclusion criteria: (1) Original article; (2) English record; (3) Published (peer reviewed); and (4) Assessment in health care practice or health care education. Finally, the imported references were subjected to the search function in EndNote as follows: Title contains ChatGPT OR Title contains Bing OR Title contains Bard. The selection of the included records was performed by the first author (Malik Sallam).

### Development of the Initial Checklist Items

Initial development of the proposed checklist began with the assessment of the methodology and results sections of the included records, a majority of which were regarded as cross-sectional descriptive studies. Then, we referred to 2 commonly used reporting and quality guidelines to proactively explore pertinent themes for the proposed checklist based on the nature of the included records: (1) STROBE (Strengthening the Reporting of Observational Studies in Epidemiology) Statement: guidelines for reporting observational studies (checklist: cross-sectional studies) and (2) CASP (Critical Appraisal Skills Programme) Checklist (CASP qualitative studies checklist) [40,41]. This facilitated the allocation of ethical considerations, including transparency, methodological rigor, and issues related to bias, in the proposed checklist. Then, the 3 authors conducted an independent content review process to identify all the possible essential themes and best practices in generative AI-based health care studies among the included records. Finally, the authors had a collaborative discussion to refine the selected themes and classify these themes into specific components relevant to the study objectives. Special attention was given to aspects of method and result reporting that were perceived to impact the quality and reproducibility of the records, as identified by the 3 authors.

### Establishing the Final Checklist Criteria

Careful examination of the included records resulted in the compilation of 3 independent lists of “pertinent themes,” which are herein defined as being critical or recurring in the reporting of results of generative AI-based studies. A thorough discussion among the authors followed to reach a consensus on the pertinent themes. Recurring themes were defined as those found in the methods of at least three separate records. Critical aspects were defined as those that would impact the conclusions of the included records as agreed by the 3 authors.

The final pertinent themes were selected based on their author-perceived significance in the quality and reproducibility of the findings. A final list of 9 themes was agreed upon by the authors as follows: (1) the “Model” of the generative AI-based tool or tools used in the included record and the explicit mention of the exact settings employed for each tool; (2) the “Evaluation” approach to assess the quality of the content generated by the generative AI-based model in terms of objectivity to reach unbiased findings and subjectivity; (3) the exact “Timing” of generative AI-based model testing and its duration; (4) the “Transparency” of data sources used to generate queries for the generative AI-based model testing, including the permission to use copyrighted content; (5) the “Range” of topics tested (single topic, multiple related topics, or various unrelated topics, as well as the breadth of intertopic and intratopic queries tested); (6) the degree of “Randomization” of topics selected to be tested to consider the potential bias; (7) the “Individual” subjective role in evaluating the content and the possibility of interrater reliability concordance or discordance; (8) the number (“Count”) of queries executed on each generative AI model entailing the sample size of queries tested; and (9) the “Specificity” of prompts used on each generative AI-based model, including the exact phrasing of each prompt and the presence of feedback and learning loops, and the “Specificity” of the language or languages used in testing, besides any other cultural issues. Thus, the final checklist was termed METRICS (Model, Evaluation, Timing, Range/Randomization, Individual factors, Count, and Specificity of prompts and language).

### Scoring the METRICS Items and Classification of the METRICS Score

Testing of the included records was performed by 2 independent raters (Malik Sallam and Mohammed Sallam) independently, with each METRICS item scored using a 5-point Likert scale as follows: 5=excellent, 4=very good, 3=good, 2=satisfactory, and 1=suboptimal. For the items that were deemed “not applicable” (eg, individual factors for studies that employed objective methods for evaluation), no score was given. The scores for the 2 raters were then averaged. The average METRICS score was calculated as the sum of average scores for each applicable item divided by 10 minus the number of items deemed not applicable.

The subjective assessment of the 2 raters was performed based on predefined criteria as a general guide. For example, if the exact dates of model queries were mentioned, the “Timing” item was scored as excellent. The count of queries was agreed to be categorized as excellent if it was more than 500, while a single case or no mention of the count was considered suboptimal. For the prompt attributes, scores were assigned based on the availability of exact prompts, explicit mention of the language used, and details of prompting. Thus, prompts and language specificity were appraised positively if the study clearly and explicitly made the exact prompts available and if there was an explicit mention of the language employed in the prompts. The evaluation method was agreed to be rated higher for objective assessments with full details and lower for subjective assessments. The explicit mention of the method of interrater reliability testing was agreed to be scored higher for the “Individual” item. Transparency was assessed based on the comprehensiveness of the data source, and the presence of full database disclosure and permission to use the data was agreed to be given an excellent score. Randomization was agreed to be scored the lowest for the absence of details and the highest for explicit detailed descriptions.

Finally, we decided to highlight the records that scored the highest for each METRICS item. The decision to take this approach was based on an attempt to refrain from providing examples for the other quality categories to avoid premature conclusions regarding the quality of the included studies owing to the preliminary pilot nature of the METRICS tool.

### Statistical and Data Analysis

The average METRICS scores were classified into distinct categories of equal weights as follows: excellent (score 4.21-5.00), very good (3.41-4.20), good (2.61-3.40), satisfactory (1.81-2.60), and suboptimal (1.00-1.80).

The Cohen κ measure was used to assess the interrater reliability by 2 independent raters. The Cohen κ measure was categorized as follows: <0.20, poor agreement; 0.21-0.40, fair agreement; 0.41-0.60, good agreement; 0.61-0.80, very good agreement; and 0.81-1.00, excellent agreement.

For statistical analysis, we used IBM SPSS Statistics for Windows, Version 26.0 (IBM Corp). A *P* value <.05 was considered significant.

## Results

### Description of the Included Studies

**Figure 1:**
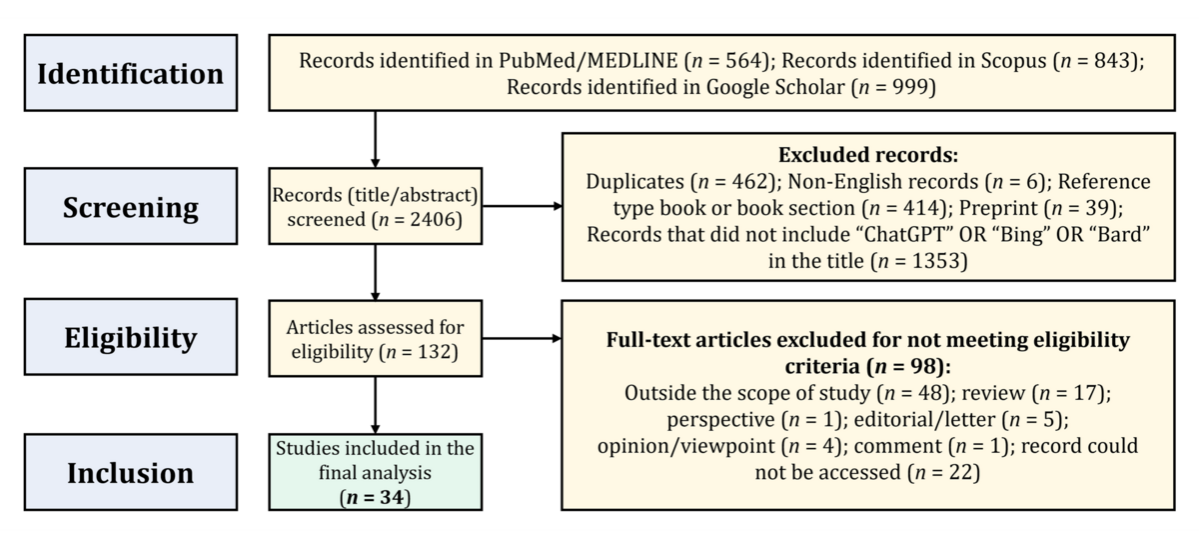
The approach for selecting articles.

A total of 34 studies were included in the final analysis that aimed to establish the METRICS criteria (Figure 1).

The most common source of records was Cureus, with 9 of the 34 records (27%), followed by BMJ Neurology Open, with 2 of the 34 records (6%). The remaining 23 records were published in 23 different journals.

### Evaluation of the Included Records Based on MED-METRICS Items

The METRICS checklist was divided into the following 3 parts: “Model” attributes, “Evaluation” approach, and features of “Data” (MED-METRICS).

The complete details of the model attributes of the included studies are presented in Table 1.

ChatGPT was tested in all the records included (34/34, 100%), followed by Google Bard (5/34, 15%) and Bing Chat (5/34, 15%). The exact dates of generative AI-based model queries were explicitly mentioned in 13 of the 34 records (38%). The count of cases or questions that were tested in the studies ranged from a single case to 2576 questions. The majority of studies (23/34, 68%) tested the AI models based on queries in the English language.

The complete details of the evaluation approach of the content generated by the AI-based models in the included studies are presented in Table 2.

Objective evaluation of the content generated by the generative AI-based model was noted in 15 of the 34 records (44%).

The complete details of the features of data used to generate queries for testing on the generative AI-based models, including the range of topics and randomization, are presented in Table 3.

Explicit mention of the randomization process was only noted in 4 of the 34 included studies (9%). Of the 34 records, 6 (18%) involved broad multidisciplinary medical exam questions (18%). Moreover, 2 studies (6%) explicitly mentioned the permission to use the data for the studies.

**Table 1:** Details of the model attributes of the included studies.

Authors	Model	Timing	Count	Specificity of the prompts and language
Al-Ashwal et al [42]	ChatGPT-3.5, ChatGPT-4, Bing, and Bard with unclear settings	One month, May 2023	255 drug-drug pairs	The exact prompts were provided in English.
Alfertshofer et al [43]	ChatGPT with unclear settings	Not provided	1800 questions	The exact prompt was used for each question. The questions were taken from United States, United Kingdom, Italian, French, Spanish, and Indian exams. A new session was used for each question.
Ali et al [44]	ChatGPT Feb 9 free version with unclear settings	Not provided	50 items	Information was provided fully in the supplementary file of the article in English.
Aljindan et al [45]	ChatGPT-4 with unclear settings	Not provided	220 questions	Initial prompting that involved role playing as a medical professional. The language was English.
Altamimi et al [46]	ChatGPT-3.5	Single day not otherwise specified	9 questions	The exact prompts were provided in English.
Baglivo et al [47]	Bing, ChatGPT, Chatsonic, Bard, and YouChat with full details of the mode and large language model, including plugins	Exact dates were provided for each model (April 12, 13, and 14, 2023, and July 13, 2023)	15 questions	Italian was used.
Biswas et al [48]	ChatGPT-3.5 with unclear settings	Exact date provided (March 16, 2023)	11 questions	The exact prompts were provided in English, with a new session for each question.
Chen et al [49]	ChatGPT-4 with unclear settings	Not provided	560 questions	The exact prompts were provided in English.
Deiana et al [50]	ChatGPT-3.5 and ChatGPT-4 with unclear settings	Not provided	11 questions	The exact prompts were not explicitly provided. English was used. A new session was used for each question. Up to three iterations were allowed for incorrect responses.
Fuchs et al [51]	ChatGPT-3 and ChatGPT-4 with unclear settings	Exact dates provided (February 19, 2023, and March 25, 2023)	60 questions	Dental medicine questions were translated from German to English, while the other questions were already present in English. Exact prompts were provided in English with prompting in 2 groups for the same questions: one group was primed with instructions, while the other was not primed. A total of 20 trials were conducted per group, and chat history was cleared after each trial.
Ghosh & Bir [52]	ChatGPT (version March 14, 2023) with unclear settings	March 14 and 16, 2023	200 questions	The exact prompts and language were not explicitly provided. The first response was taken as final, and the option of “regenerate response” was not used.
Giannos [53]	ChatGPT-3 and ChatGPT-4 with unclear settings	Not provided	69 questions	Not explicitly provided.
Gobira et al [54]	ChatGPT-4 with unclear settings	Not provided	125 questions	Portuguese was used.
Grewal et al [55]	ChatGPT-4 with unclear settings	The first week of May 2023	Not clear	The exact prompts were provided in English. One follow-up prompt was used for enhancement of some prompts.
Guerra et al [56]	ChatGPT-4 with unclear settings	Not provided	591 questions	The exact prompts were provided, while the language was not explicitly provided.
Hamed et al [57]	ChatGPT-4 with unclear settings	Not provided	Not clear	The exact prompts and language were not explicitly provided. Different prompts were tried to identify the most suitable.
Hoch et al [58]	ChatGPT (May 3rd version) with unclear settings	May 5 and 7, 2023	2576 questions	The exact prompts were provided, while the language was not explicitly provided.
Juhi et al [59]	ChatGPT with unclear settings	February 20, 2023, to March 5, 2023	40 drug-drug pairs	The exact prompts were provided in English.
Kuang et al [60]	ChatGPT with unclear settings	Not provided	Not clear	The exact prompts were not explicitly provided. English was used.
Kumari et al [61]	ChatGPT-3.5, Bard, and Bing with unclear settings	July 30, 2023	50 questions	The exact prompts were not explicitly provided. English was used.
Kung et al [62]	ChatGPT-3.5 and ChatGPT-4 with unclear settings	July 2023	215 questions	Not clear
Lai et al [63]	ChatGPT-4 (May 24; Version 3.5) with unclear settings	Not provided	200 questions	The exact prompts and language were not explicitly provided. Three attempts to answer the complete set of questions over 3 weeks (once per week), with a new session for each question.
Lyu et al [64]	ChatGPT with unclear settings	Mid-February 2023	Not clear	The exact prompts were provided in English.
Moise et al [65]	ChatGPT-3.5 with unclear settings	Not provided	23 questions	The exact prompts were provided in English, with a new session for each question.
Oca et al [66]	ChatGPT, Bing, and Bard with unclear settings	April 11, 2023	20 queries for each model	The exact prompts were provided in English.
Oztermeli & Oztermeli [67]	ChatGPT-3.5 with unclear settings	Not provided	1177 questions	The exact prompts were not explicitly provided. Turkish was used, with a new session for each question.
Pugliese et al [68]	ChatGPT with unclear settings	March 25, 2023	15 questions	The exact prompts were provided in English, with a new session for each question.
Sallam et al [69]	ChatGPT (default model) with unclear settings	February 25, 2023	Not provided	The exact prompts were provided in English.
Seth et al [70]	ChatGPT-3.5, Bard, and Bing AI	Not provided	6 questions	The exact prompts were provided in English.
Suthar et al [71]	ChatGPT-4 with unclear settings	Not provided	140 cases	The exact prompts were not explicitly provided. English was used.
Walker et al [72]	ChatGPT-4 with unclear settings	Not provided	5 cases	The exact prompts were not explicitly provided. English was used, with a new session for each question.
Wang et al [73]	ChatGPT-3.5 and ChatGPT-4 with unclear settings	February 14, 2023, for ChatGPT-3.5 and May 14-16, 2023, for ChatGPT-4	300 questions	The exact prompts were provided. Chinese and English were used. The prompts were enhanced though role play.
Wang et al [74]	ChatGPT-3.5 with unclear settings	March 5-10, 2023	Not clear	Chinese (Mandarin) and English were used. Examples of prompts were provided.
Zhou et al [75]	ChatGPT-3.5 with unclear settings	April 24-25, 2023	Single case and multiple poll questions	The exact prompts were provided in English.

**Table 2:** Classification based on the evaluation approach of the content generated by the artificial intelligence–based models.

Authors	Evaluation of performance	Individual role and interrater reliability
Al-Ashwal et al [42]	Objective via 2 different clinical reference tools	Not applicable
Alfertshofer et al [43]	Objective based on the key answers, with the questions screened independently by 4 investigators	Not applicable
Ali et al [44]	Objective for multiple-choice questions and true or false questions, and subjective for short-answer and assay questions	Assessment by 2 assessors independently with intraclass correlation coefficient for agreement
Aljindan et al [45]	Objective based on key answers and historical performance metrics	Not applicable
Altamimi et al [46]	Subjective	Not clear; Assessment for accuracy, informativeness, and accessibility by clinical toxicologists and emergency medicine physicians
Baglivo et al [47]	Objective based on key answers and comparison with 5th year medical students’ performance	Not applicable
Biswas et al [48]	Subjective by a 5-member team of optometry teaching and expert staff with over 100 years of clinical and academic experience between them; Independent evaluation on a 5-point Likert scale ranging from very poor to very good	The median scores across raters for each response were studied; The score represented rater consensus, while the score variance represented disagreements between the raters
Chen et al [49]	Objective based on key answers	Not applicable
Deiana et al [50]	Subjective based on qualitative assessment of correctness, clarity, and exhaustiveness; Each response rated using a 4-point Likert scale scored from strongly disagree to strongly agree	Independent assessment by 2 raters with experience in vaccination and health communication topics
Fuchs et al [51]	Objective based on key answers	Not applicable
Ghosh & Bir [52]	Objective based on key answers; Subjectivity by raters’ assessment	Scoring by 2 assessors on a scale of 0 to 5, with 0 being incorrect and 5 being fully correct, based on a preselected answer key
Giannos [53]	Objective based on key answers	Not applicable
Gobira et al [54]	Objective based on key answers, with an element of subjectivity through classifying the responses as adequate, inadequate, or indeterminate	Two raters independently scored the accuracy; After individual evaluations, the raters performed a third assessment to reach a consensus on the questions with differing results
Grewal et al [55]	Not clear	Not clear
Guerra et al [56]	Subjective through comparison with the results of a previous study on the average performance of users, and a cohort of medical students and neurosurgery residents	Not applicable
Hamed et al [57]	Subjective	Not clear
Hoch et al [58]	Objective based on key answers	Not applicable
Juhi et al [59]	Subjective and the use of Stockley’s Drug Interactions Pocket Companion 2015 as a reference key	Two raters reached a consensus for categorizing the output
Kuang et al [60]	Subjective	Not clear
Kumari et al [61]	Subjective; Content validity checked by 2 experts of curriculum design	Three independent raters scored content based on their correctness, with an accuracy score ranging from 1 to 5
Kung et al [62]	Objective based on key answers	Not applicable
Lai et al [63]	Objective based on key answers	Not applicable
Lyu et al [64]	Subjective	Two experienced radiologists (with 21 and 8 years of experience) evaluated the quality of the ChatGPT responses
Moise et al [65]	Subjective through comparison with the latest American Academy of Otolaryngology–Head and Neck Surgery Foundation Clinical Practice Guideline: Tympanostomy Tubes in Children (Update)	Two independent raters evaluated the output; The interrater reliability was assessed using the Cohen κ test; To confirm consensus, responses were reviewed by the senior author
Oca et al [66]	Not clear	Not clear
Oztermeli & Oztermeli [67]	Objective based on key answers	Not applicable
Pugliese et al [68]	Subjective using the Likert scale for accuracy, completeness, and comprehensiveness	Multirater: 10 key opinion leaders in nonalcoholic fatty liver disease and 1 nonphysician with expertise in patient advocacy in liver disease independently rating the AI^a^ content
Sallam et al [69]	Subjective based on correctness, clarity, and conciseness	Fleiss multirater κ
Seth et al [70]	Subjective through comparison with the current health care guidelines for rhinoplasty, and evaluation by a panel of plastic surgeons through a Likert scale to assess the readability and complexity of the text and the education level required for understanding, and the modified DISCERN^b^ score	Not clear
Suthar et al [71]	Subjective by 3 fellowship-trained neuroradiologists, using a 5-point Likert scale, with 1 indicating “highly improbable” and 5 indicating “highly probable”	Not applicable
Walker et al [72]	Modified EQIP^c^ Tool with comparison with UK National Institute for Health and Care Excellence guidelines for gallstone disease, pancreatitis, liver cirrhosis, or portal hypertension, and the European Association for Study of the Liver guidelines	All answers were assessed by 2 authors independently, and in case of a contradictory result, resolution was achieved by consensus; The process was repeated 3 times per EQIP item; Wrong or out of context answers, known as “AI hallucinations,” were recorded
Wang et al [73]	Subjective	Unclear
Wang et al [74]	Objective based on key answers	Not applicable
Zhou et al [75]	Subjective	Unclear

^a^AI: artificial intelligence.

^b^DISCERN is an instrument for judging the quality of written consumer health information on treatment choices.

^c^EQIP: Ensuring Quality Information for Patients.

**Table 3:** Classification of the included studies based on the features of data used to generate queries for testing on the generative artificial intelligence–based models.

Authors	Transparency	Range	Randomization
Al-Ashwal et al [42]	Full description using 2 tools for assessment: Micromedex, a subscription-based drug-drug interaction screening tool, and Drugs.com, a free database	Narrow; Drug-drug interaction prediction	Nonrandom; Purposeful selection of the drugs by a clinical pharmacist; 5 drugs paired with the top 51 prescribed drugs
Alfertshofer et al [43]	Full description using the question bank AMBOSS, with official permission for the use of the AMBOSS USMLE step 2CK practice question bank for research purposes granted by AMBOSS	Broad	Randomly extracted
Ali et al [44]	Developed by the researchers and reviewed by a panel of experienced academics for accuracy, clarity of language, relevance, and agreement on correct answers; Evaluation of face validity, accuracy, and suitability for undergraduate dental students	Narrow intersubject (dentistry); Broad intrasubject in restorative dentistry, periodontics, fixed prosthodontics, removable prosthodontics, endodontics, pedodontics, orthodontics, preventive dentistry, oral surgery, and oral medicine	Not clear
Aljindan et al [45]	Saudi Medical Licensing Exam questions extracted from the subscription CanadaQBank website	Broad in medicine, with 30% of the questions from medicine, 25% from obstetrics and gynecology, 25% from pediatrics, and the remaining 20% from surgery	Randomized through 4 researchers to ensure comprehensive coverage of questions and eliminate potential bias in question selection
Altamimi et al [46]	Snakebite management information guidelines derived from the World Health Organization, Centers for Disease Control and Prevention, and the clinical literature	Narrow	Not clear
Baglivo et al [47]	The Italian National Medical Residency test	Narrow; Vaccination-related questions from the Italian National Medical Residency Test	Not clear
Biswas et al [48]	Constructed based on the frequently asked questions on the myopia webpage of the Association of British Dispensing Opticians and the College of Optometrists	Narrow involving 9 categories: 1 each for disease summary, cause, symptom, onset, prevention, complication, natural history of untreated myopia, and prognosis, and 3 involving treatments	Not clear
Chen et al [49]	BoardVitals, which is an online question bank accredited by the Accreditation Council for Continuing Medical Education	Neurology-based; Broad intrasubject: basic neuroscience; behavioral, cognitive, psychiatry; cerebrovascular; child neurology; congenital; cranial nerves; critical care; demyelinating disorders; epilepsy and seizures; ethics; genetic; headache; imaging or diagnostic studies; movement disorders; neuro-ophthalmology; neuro-otology; neuroinfectious disease; neurologic complications of systemic disease; neuromuscular; neurotoxicology, nutrition, metabolic; oncology; pain; pharmacology; pregnancy; sleep; and trauma	Not clear
Deiana et al [50]	Questions concerning vaccine myths and misconceptions by the World Health Organization	Narrow on vaccine myths and misconceptions	Not clear
Fuchs et al [51]	Digital platform self-assessment questions tailored for dental and medical students at the University of Bern’s Institute for Medical Education	Broad with multiple-choice questions designed for dental students preparing for the Swiss Federal Licensing Examination in Dental Medicine, and allergists and immunologists preparing for the European Examination in Allergy and Clinical Immunology	Not clear
Ghosh & Bir [52]	Department question bank, which is a compilation of first and second semester questions from various medical universities across India	Biochemistry	Random without details of randomization
Giannos [53]	Specialty Certificate Examination Neurology Web Questions bank	Neurology and neuroscience	Not clear
Gobira et al [54]	National Brazilian Examination for Revalidation of Medical Diplomas issued by Foreign Higher Education Institutions (Revalida)	Preventive medicine, gynecology and obstetrics, surgery, internal medicine, and pediatrics	Not clear
Grewal et al [55]	Not clear	Radiology	Not clear
Guerra et al [56]	Questions released by the Congress of Neurological Surgeons in the self-assessment neurosurgery exam	Neurosurgery across 7 subspecialties: tumor, cerebrovascular, trauma, spine, functional, pediatrics, and pain or nerve	Not clear
Hamed et al [57]	Guidelines adapted from Diabetes Canada Clinical Practice Guidelines Expert Committee, the Royal Australian College of General Practitioners, Australian Diabetes Society position statement, and the Joint British Diabetes Societies	Management of diabetic ketoacidosis	Not clear
Hoch et al [58]	Question database of an online learning platform funded by the German Society of Oto-Rhino-Laryngology, Head and Neck Surgery	Otolaryngology with a range of 15 distinct otolaryngology subspecialties, including allergology, audiology, ear, nose and throat, tumors, face and neck, inner ear and skull base, larynx, middle ear, oral cavity and pharynx, nose and sinuses, phoniatrics, salivary glands, sleep medicine, vestibular system, and legal aspects	Not clear
Juhi et al [59]	A list of drug-drug interactions from previously published research	Narrow on drug-drug interaction	Not clear
Kuang et al [60]	Not clear	Neurosurgery	Not clear
Kumari et al [61]	Designed by experts in hematology-related cases	Hematology with the following intrasubject aspects: case solving, laboratory calculations, disease interpretations, and other relevant aspects of hematology	Not clear
Kung et al [62]	Orthopedic In-Training Examination	Orthopedics	Not clear
Lai et al [63]	The United Kingdom Medical Licensing Assessment, which is a newly derived national undergraduate medical exit examination	Broad in medicine with the following aspects: acute and emergency, cancer, cardiovascular, child health, clinical hematology, ear, nose and throat, endocrine and metabolic, gastrointestinal including liver, general practice and primary health care, genetics and genomics, infection, medical ethics and law, medicine of older adults, mental health, musculoskeletal, neuroscience obstetrics and gynecology, ophthalmology, palliative and end of life care, perioperative medicine and anesthesia, renal and urology, respiratory, sexual health, social and population health, and surgery	Not clear
Lyu et al [64]	Chest computed tomography and brain magnetic resonance imaging screening reports collected from the Atrium Health Wake Forest Baptist clinical database	Radiology	Not clear
Moise et al [65]	Statements published in the latest American Academy of Otolaryngology–Head and Neck Surgery Foundation Clinical Practice Guideline: Tympanostomy Tubes in Children (Update)	Narrow involving otolaryngology	Not clear
Oca et al [66]	Not clear	Narrow involving only queries on accurate recommendation of close ophthalmologists	Not clear
Oztermeli & Oztermeli [67]	Turkish medical specialty exam, prepared by the Student Selection and Placement Center	Broad: basic sciences including anatomy, physiology-histology-embryology, biochemistry, microbiology, pathology, and pharmacology; clinical including internal medicine, pediatrics, general surgery, obstetrics and gynecology, neurology, neurosurgery, psychiatry, public health, dermatology, radiology, nuclear medicine, otolaryngology, ophthalmology, orthopedics, physical medicine and rehabilitation, urology, pediatric surgery, cardiovascular surgery, thoracic surgery, plastic surgery, anesthesiology and reanimation, and emergency medicine	Not clear
Pugliese et al [68]	Expert selection of 15 questions commonly asked by patients with nonalcoholic fatty liver disease	Narrow involving nonalcoholic fatty liver disease aspects	Not clear
Sallam et al [69]	Panel discussion of experts in health care education	Broad on health care education, medical, dental, pharmacy, and public health	Not clear
Seth et al [70]	Devised by 3 fellows of the Royal Australasian College of Surgeons, with experience in performing rhinoplasty and expertise in facial reconstructive surgery	Narrow involving technical aspects of rhinoplasty	Not clear
Suthar et al [71]	Quizzes from the Case of the Month feature of the American Journal of Neuroradiology	Narrow involving radiology	Not clear
Walker et al [72]	Devised based on the Global Burden of Disease tool	Narrow involving benign and malignant hepatopancreaticobiliary-related conditions	Not clear
Wang et al [73]	Medical Exam Help. A total of 10 inpatient and 10 outpatient medical records to form a collection of Chinese medical records after desensitization	Clinical medicine, basic medicine, medical humanities, and relevant laws	Not clear
Wang et al [74]	The Taiwanese Senior Professional and Technical Examinations for Pharmacists downloaded from the Ministry of Examination website	Broad involving pharmacology and pharmaceutical chemistry, pharmaceutical analysis and pharmacognosy (including Chinese medicine), pharmaceutics and biopharmaceutics, dispensing pharmacy and clinical pharmacy, therapeutics, pharmacy administration, and pharmacy law	Not clear
Zhou et al [75]	A single clinical case from OrthoBullets, a global clinical collaboration platform for orthopedic surgeons; Written permission to use their clinical case report	Very narrow involving a single orthopedic case	Not clear

### Examples of Optimal Reporting of Each Criterion Within the METRICS Checklist

The records with the highest scores for each METRICS item, as determined by the average subjective interrater assessments, are shown in Table 4.

**Table 4:** Included records that had the highest METRICS score per item.

Item	Issues considered in each item	Excellent or very good reporting examples
#1 Model	What is the model of the generative AI^a^ tool used for generating content, and what are the exact settings for each tool?	Baglivo et al [47]: Bing, ChatGPT, Chatsonic, Bard, and YouChat, with full details of the mode and large language model, including plugins
#2 Evaluation	What is the exact approach used to evaluate the content generated by the generative AI-based model and is it an objective or subjective evaluation?	Al-Ashwal et al [42]: Objective via 2 different clinical reference tools; Alfertshofer et al [43]: Objective based on key answers, with the questions screened independently by 4 investigators; Ali et al [44]: Objective for multiple-choice questions and true or false questions, and subjective for short-answer and assay questions; Aljindan et al [45]: Objective based on key answers and historical performance metrics; and Baglivo et al [47]: Objective based on key answers and comparison with 5th year medical students’ performance
#3a Timing	When is the generative AI model tested exactly and what are the duration and timing of testing?	Baglivo et al [47]; Biswas et al [48]; Fuchs et al [51]; Ghosh & Bir [52]; Hoch et al [58]; Juhi et al [59]; Kumari et al [61]; Kung et al [62]; Oca et al [66]; Pugliese et al [68]; Sallam et al [69]; Wang et al [73]; and Zhou et al [75]
#3b Transparency	How transparent are the data sources used to generate queries for the generative AI-based model?	Alfertshofer et al [43]
#4a Range	What is the range of topics tested and are they intersubject or intrasubject with variability in different subjects?	Ali et al [44]; Chen et al [49]; Hoch et al [58]; and Wang et al [73]
#4b Randomization	Was the process of selecting the topics to be tested on the generative AI-based model randomized?	Alfertshofer et al [43] and Aljindan et al [45]
#5 Individual	Is there any individual subjective involvement in generative AI content evaluation? If so, did the authors describe the details in full?	Ali et al [44] and Moise et al [65]
#6 Count	What is the count of queries executed (sample size)?	Alfertshofer et al [43]; Chen et al [49]; Guerra et al [56]; Hoch et al [58]; and Oztermeli & Oztermeli [67]
#7 Specificity of the prompt or language	How specific are the exact prompts used? Were those exact prompts provided fully? Did the authors consider the feedback and learning loops? How specific are the language and cultural issues considered in the generative AI model?	Alfertshofer et al [43]; Biswas et al [48]; Fuchs et al [51]; Grewal et al [55]; Wang et al [73]; Moise et al [65]; and Pugliese et al [68]

^a^AI: artificial intelligence.

### Interrater Assessment of the Included Records Based on METRICS Scores

The overall mean METRICS score was 3.0 (SD 0.58). For each item, the κ interrater reliability ranged from 0.558 to 0.962 (*P*<.001 for the 9 tested items), indicating good to excellent agreement (Table 5).

With classification per item, the highest average METRICS score was recorded for the “Model” item, followed by the “Specificity” item, while the lowest scores were recorded for the “Randomization” item (classified as suboptimal) and “Individual factors” item (classified as satisfactory) (Table 5).

**Table 5:** Interrater reliability per METRICS item.

METRICS^a^ item	Score			Quality		Cohen κ		Asymptotic standard error		Approximate T		*P* value
	Mean^b^ (SD)	Range										
Model	3.72 (0.58)	2.5-5.0	Very good		0.820		0.090		6.044		<.001	
Timing	2.90 (1.93)	1.0-5.0	Good		0.853		0.076		6.565		<.001	
Count	3.04 (1.32)	1.0-5.0	Good		0.962		0.037		10.675		<.001	
Specificity of prompts and language	3.44 (1.25)	1.0-5.0	Very good		0.765		0.086		8.083		<.001	
Evaluation	3.31 (1.16)	1.0-5.0	Good		0.885		0.063		9.668		<.001	
Individual factors	2.50 (1.42)	1.0-5.0	Satisfactory		0.865		0.087		6.860		<.001	
Transparency	3.24 (1.01)	1.0-5.0	Good		0.558		0.112		5.375		<.001	
Range	3.24 (1.07)	2.0-5.0	Good		0.836		0.076		8.102		<.001	
Randomization	1.31 (0.87)	1.0-4.0	Suboptimal		0.728		0.135		5.987		<.001	
Overall	3.01 (0.58)	1.5-4.1	Good		0.381		0.086		10.093		<.001	

^a^METRICS: Model, Evaluation, Timing, Range/Randomization, Individual factors, Count, and Specificity of prompts and language.

^b^The mean scores represent the results of evaluating the included studies averaged for the 2 rater scores.

## Discussion

### Principal Findings

The interpretation and synthesis of credible scientific evidence based on studies evaluating commonly used generative AI-based conversational models (eg, ChatGPT, Bing, and Bard) can be challenging. This is related to the discernible variability in the methods used for the evaluation of such models, as well as the varying styles of reporting. Such variability is fathomable considering the emerging nature of this research field with less than a year of reporting at the time of writing. Therefore, a standardized framework to guide the design of such studies and to delineate the best reporting practices can be beneficial, since rigorous methodology and clear reporting of findings are key attributes of science to reach reliable conclusions with real-world implications.

In this study, a preliminary checklist referred to as “METRICS” was formulated, which can help researchers aspiring to test the performance of generative AI-based models in various aspects of health care education and practice. It is crucial to explicitly state that the proposed METRICS checklist in this study cannot be claimed to be comprehensive or flawless. Nevertheless, this checklist could provide a solid base for future studies and much needed efforts aiming to standardize the reporting of AI-based studies in health care.

The principal finding of this study was the establishment of 9 key themes that are recommended to be considered in the design, testing, and reporting of generative AI-based models in research, particularly in the health care domain. These features included the model of the generative AI tool, evaluation approach, timing of testing and transparency, range of topics tested and randomization of queries, individual factors in the design and assessment, count of queries, and specific prompts and languages used. The relevance of these themes in the design and reporting of generative AI-based model content testing can be illustrated as follows.

First, the variability in generative AI model types used to conduct queries and the variability in settings pose significant challenges for cross-study comparisons. The significant impact of generative AI models on the resultant output is related to the distinct architectures and capabilities of various generative AI models, with expected variability in the performance and quality of the content generated by generative AI [76]. Additionally, various options to configure the models further affect the content generated by generative AI. Consequently, it is important to consider these variations when evaluating research using different generative AI models [77-81]. These issues can be illustrated clearly in the records included in this study that conducted contemporary analysis of at least two models. For example, Al-Ashwal et al [42] showed that Bing had the highest accuracy and specificity for predicting drug-drug interaction, outperforming Bard, ChatGPT-3.5, and ChatGPT-4. Moreover, Baglivo et al [47] showed not only intermodel variability but also intramodel variability in performance in the domain of public health. Additionally, in the context of providing information on rhinoplasty, Seth et al [70] showed that this intermodel variability in performance was the most comprehensible with the content of Bard, followed by ChatGPT and Bing.

Second, the continuous updating of generative AI models introduces significant temporal variability, which would influence the comparability of studies conducted at different times. The updates of generative AI models enhance their capabilities and performance [82]. Consequently, this temporal variability can lead to inconsistencies in synthesizing evidence, as the same model may demonstrate different outputs over time. Therefore, when analyzing or comparing studies involving AI models, it is crucial to consider the specific version and state of the model at the time of each study to accurately interpret and compare results. In this context, it is important to conduct future longitudinal studies to discern the exact effect of changes in performance of commonly used generative AI models over time.

Third, the count of queries in the evaluation of generative AI models was identified among the pertinent themes of assessment. This appears understandable since studies employing a larger number of queries can provide a more comprehensive evaluation of the tested model. An extensive number of queries can reveal minor weaknesses, despite the difficulty to establish what constitutes an “extensive” number of queries of the minimum number of queries needed to reveal the real performance of a generative AI model in a particular topic. In this study, the number of queries varied from a single case to more than 2500 questions, showing the need for standardization and establishing a clear guide on the number of queries deemed suitable [58,75].

Fourth, a key theme identified in this study was the nature and language of the prompts used to conduct the studies. This involved the imperative of explicitly stating the used prompts and the language in which they were framed. The exact prompting approach and the presence of cultural and linguistic biases appear to be critical factors that can influence the quality of content generated by generative AI-based models [83]. Slight differences in wording or context in the prompt used to generate the generative AI content can lead to recognizable differences in the content generated [36,84,85]. Additionally, feedback mechanisms and learning loops that allow generative AI-based models to learn from interactions can change the model performance for the same query, which might not be consistently accounted for in all studies. These minor variations in prompts across different studies can also complicate the synthesis of evidence, highlighting the need for standardizing such an aspect. Additionally, as highlighted above, generative AI-based models may exhibit biases based on their training data, affecting performance across various cultural or linguistic contexts [86-88]. Thus, studies conducted in different regions or involving various languages might yield varying results. In this study, we found that a majority of the included records tested generative AI-based models using the English language, highlighting the need for more studies on other languages to reveal the possible variability in performance based on language. Comparative studies involving multiple languages can reveal such inconsistencies, for example, the study by Wang et al [74]. In the aforementioned study assessing ChatGPT performance in the Taiwanese pharmacist licensing exam, the performance in the English test was better than that in the Chinese test across all tested subjects [74]. Another comprehensive study by Alfertshofer et al [43] that assessed the performance of ChatGPT on 6 different national medical licensing exams highlighted the variability in performance per country and language. Based on the previous points, more studies that encompass diverse language and cultural perspectives are essential to assess and address possible cultural and language biases in generative AI-based models. Additionally, the design of generative AI-based models trained on a more diverse set of languages and cultural contexts is important to ensure that the training data sets are representative of different linguistic groups, which is of paramount importance in health care. Furthermore, cross-cultural validation studies appear valuable for understanding the performance of generative AI-based models in various language and cultural settings. These approaches could enhance the broad applicability of generative AI-based models in health care to ensure the fair distribution of the benefits of generative AI technologies.

Fifth, an important theme highlighted in this study was the approach of evaluating the content generated by generative AI-based models. Variable methods of assessment can introduce a discernible methodological variability. Specifically, the use of objective assessment ensures consistency in assessment. On the other hand, subjective assessment, even by experts, can vary despite the professional judgment and deep understanding provided by such an expert opinion [89].

Similarly, the number and expertise of evaluators or raters involved in constructing and evaluating the generative AI-based studies were identified as a pertinent theme in this study [90,91]. Variations in rater numbers across studies can lead to inconsistencies in synthesized evidence [68,69,92]. Additionally, the method used to establish agreement (eg, kappa statistics and consensus meetings) might differ in various studies, affecting the comparability of results.

Finally, data-pertinent issues were identified as key themes in this study. These involved the need for full transparency regarding the sources of data used to create the queries (eg, question banks, credible national and international guidelines, clinical reports, etc) [93,94]. Additionally, ethical considerations, such as consent to use copyrighted material and consent or anonymization of the clinical data, should be carefully stated in the evaluation studies of AI-based models. An important aspect that appeared suboptimal in the majority of included records was randomization to reduce or eliminate potential bias in query selection. Thus, this important issue should be addressed in future studies to allow unbiased evaluation of the content of generative AI-based models. Another important aspect is the need to carefully select the topics to be tested, which can belong to a broad domain (eg, medical examination) or a narrow domain (eg, a particular specialty) [95-97]. A comprehensive description of topics is essential to reveal subtle differences in generative AI performance across various domains. Biased query coverage per topic may result in unreliable conclusions regarding generative AI model performance.

The value of the METRICS checklist in guiding researchers toward more rigorous design and transparent reporting of health care studies assessing the performance of generative AI-based models can be highlighted as follows. Two studies exemplified the practical utility of the METRICS checklist (presented in its preprint form) across different research scenarios (health care education and health care practice) [98]. The first study conducted a detailed assessment of ChatGPT-3.5 performance in medical microbiology multiple choice questions compared with dental students [99]. By applying the METRICS checklist retrospectively, the study quality was delineated, including the identification of potential limitations, such as the absence of randomization, thus offering a more critical evaluation of the research design [99].

The second study investigating the performance of both ChatGPT-3.5 and ChatGPT-4 in the context of diagnostic microbiology case scenarios was conceived based on the METRICS checklist [100]. The prospective incorporation of the METRICS checklist was particularly instrumental in refining the study design and the reporting of results [100].

Thus, the aforementioned studies emphasize the effectiveness of the METRICS checklist as a tool to standardize research practice in a rapidly growing research field. The real-world application of the METRICS checklist has been valuable in identifying potential research limitations and in enhancing the overall structure and clarity of the reporting of results. These studies also demonstrate the value of the METRICS checklist for guiding researchers toward more rigorous design and transparent reporting of generative AI-based studies in health care [99,100].

### Limitations

It is crucial to explicitly mention the need for careful interpretation of the findings based on the following limitations. First, the initial search process involved the broad term “artificial intelligence” and was conducted by a single author, which may have inadvertently resulted in missing relevant references. The record selection process was further limited by the screening of record titles for only 3 generative AI models (ChatGPT, Bing, and Bard) using the EndNote search function. Additionally, the reliance on including published English records, indexed in Scopus, PubMed, or Google Scholar, could raise concerns about potential selection bias and the exclusion of relevant studies. However, it is important to consider this limitation in light of the context of our study, which represents a preliminary report that needs to be validated by future comprehensive and exhaustive studies. Second, it is important to acknowledge that a few pertinent themes could have been overlooked despite our attempt to achieve a thorough analysis, given the limited number of authors. Therefore, future follow-up studies can benefit from inclusion of authors with diverse backgrounds, including different health care disciplines, computer scientists, researchers in the field of human-AI interaction, and AI developers. Additionally, the subjective nature of the pertinent theme selection can be considered as another important caveat in this study. This shortcoming extended to involve the raters’ subjective assessments in assigning different METRICS scores. Moreover, the equal weight given to each item of the checklist in the METRICS score might not be a suitable approach, given the possibility of varying importance of each component. Thus, future comprehensive studies should focus on the relative importance of each METRICS component and its possible impact on the reporting of generative AI model performance. Third, the focus on a few specific generative AI-based conversational models (ie, ChatGPT, Bing, and Bard) can potentially overlook the nuanced aspects of other generative AI models. Nevertheless, our approach was justified by the popularity and widespread use of these particular generative AI-based models. However, it is important for future studies to expand the scope to include models, such as Llama or Claude, which could provide a more comprehensive evaluation of the utility of the METRICS checklist. Lastly, we fully and unequivocally acknowledge that the METRICS checklist is preliminary and needs further verification to ensure its valid applicability.

### Future Perspectives

The METRICS checklist proposed in this study could be a helpful step toward establishing useful guidelines to design and report the findings of generative AI-based studies. The integration of generative AI models in health care education and practice necessitates a collaborative approach involving health care professionals, researchers, and AI developers. Synthesis of evidence with critical appraisal of the quality of each element in the METRICS checklist is recommended for continuous enhancement of AI output, which would result in successful implementation of AI models in health care while avoiding possible concerns. Regular multidisciplinary efforts and iterative revisions are recommended to ensure that the METRICS checklist properly reflects its original intended purpose of improving the quality of study design and result reporting in this swiftly evolving research field. Future studies would benefit from expanding the scope of literature review and data inclusion, with the incorporation of a wider range of databases, languages, and AI models. This is crucial for reaching the ultimate aim of standardization of the design and reporting of generative AI-based studies.

### Conclusions

The newly devised “METRICS” checklist may represent a key initial step to motivate the standardization of reporting of generative AI-based studies in health care education and practice. Additionally, the establishment of this algorithm can motivate collaborative efforts to develop universally accepted reporting guidelines for the design and reporting of results of generative AI-based studies. In turn, this can enhance the comparability and reliability of evidence synthesis from these studies. The METRICS checklist, as presented by the findings of this study, can help to elucidate the strengths and limitations of the content generated by generative AI-based models, guiding their future development and application. The standardization offered by the METRICS checklist can be important to ensure the reporting of reliable and replicable results. Subsequently, this can result in the exploitation of the promising potential of generative AI-based models in health care while avoiding its possible concerns. The METRICS checklist could mark the significant progress in the evolving research field. Nevertheless, there is room for refinement through revisions and updates to verify its validity.

## Abbreviations

AI: artificial intelligence
CASP: Critical Appraisal Skills Programme
METRICS: Model, Evaluation, Timing, Range/Randomization, Individual factors, Count, and Specificity of prompts and language

## Data Availability

The data used in this study are available upon request from the corresponding author (Malik Sallam).
